# Reducing the diversity of allogeneic transplant protocols in the UK through a BSBMT Anthony Nolan Protocol Harmonization Initiative

**DOI:** 10.1038/s41409-020-0870-0

**Published:** 2020-03-24

**Authors:** Chloe Anthias, Jane Apperley, Adrian Bloor, Jennifer Byrne, Matthew Collin, Charles Crawley, Charles Craddock, Damian Finnegan, Maria Gilleece, John Gribben, Ann Hunter, Hannah Hunter, Mickey Koh, Stephen Mackinnon, Ram Malladi, David Marks, Grant McQuaker, Manos Nikolousis, Kim Orchard, Jiri Pavlu, Andrew Peniket, Mike Potter, Victoria Potter, Stephen Robinson, Nigel Russell, Rahuman Salim, John Snowden, Kirsty Thomson, Eleni Tholouli, Keith Wilson

**Affiliations:** 1 The Royal Marsden NHS Foundation Trust, London, UK; 2 Hammersmith Hospital, Imperial College Healthcare NHS Trust, London, UK; 3 The Christie NHS Foundation Trust, Manchester, UK; 4 Nottingham City Hospital, Nottingham University Hospitals NHS Trust, Nottingham, UK; 5 Northern Center for Cancer Care, Newcastle upon Tyne Hospitals NHS Foundation Trust, Newcastle upon Tyne, UK; 6 Addenbrooke’s Hospital, Cambridge University Hospitals NHS Foundation Trust, Cambridge, UK; 7 Queen Elizabeth Hospital, University Hospitals Birmingham NHS Foundation Trust, Birmingham, UK; 8 Belfast City Hospital, Belfast, UK; 9 St James’s University Hospital, The Leeds Teaching Hospitals NHS Trust, Leeds, UK; 10 Barts Cancer Center, Barts Health NHS Trust, Leeds, UK; 11 Leicester Royal Infirmary, University Hospitals of Leicester NHS Trust, Leeds, UK; 12 Derriford Hospital, University Hospitals Plymouth NHS Trust, Plymouth, UK; 13 St George’s University Hospitals NHS Foundation Trust, London, UK; 14 University College London Hospitals NHS Foundation Trust, London, UK; 15 Bristol Haematology and Oncology Center, University Hospitals Bristol NHS Foundation Trust, Bristol, UK; 16 The Beatson West of Scotland Cancer Center, Glasgow, UK; 17 Heartlands Hospital, University Hospitals Birmingham NHS Foundation Trust, Birmingham, UK; 18 University Hospital Southampton, NHS Foundation Trust, Southampton, UK; 19 Oxford University Hospitals NHS Foundation Trust, Oxford, UK; 20 King’s College Hospital NHS Foundation Trust, London, UK; 21 Royal Liverpool and Broadgreen University Hospital NHS Trust, Liverpool, UK; 22 Royal Hallamshire Hospital, Sheffield Teaching Hospitals NHS Foundation Trust, Sheffield, UK; 23 Manchester Royal Infirmary, Manchester University NHS Foundation Trust, Leeds, UK; 24 University Hospital of Wales, Cardiff and Vale University Health Board, Cardiff, UK

**Keywords:** Translational research, Haematological cancer

## To the Editor:

The majority of bone marrow transplants (BMT) are conditioned with reduced intensity protocols, greatly expanding the range of eligible patients and disease indications [1]. However, protocol diversity now presents a major obstacle to evidence-based assessments of the effectiveness of BMT in pathways of care. Protocol diversity also has a potential negative impact on the credibility of BMT as a therapeutic option in the view of other healthcare professionals, trial investigators, and healthcare purchasers. Here, we report the findings of a British Society of Bone Marrow Transplantation and Anthony Nolan Protocol Harmonization Initiative that evaluated the diversity of reduced intensity BMT protocols in use at all 25 UK transplant centers. The aim of the survey was to identify opportunities to achieve a more harmonized approach in order to facilitate future registry based and prospective clinical studies.

Reduced intensity conditioning (RIC) protocols for BMT have proliferated and diversified enormously, since the late 1990s [1]. A strong impetus to innovate has resulted in empirical adoption of many different protocols unsupported by trial evidence. While fludarabine has become almost universally established as a useful agent, it has been combined with a wide range and dosing schedules of chemotherapy and radiotherapy. Variation in T-cell depletion strategies has added further diversity. Alemtuzumab, effective for the transplantation of unrelated donors, is now frequently incorporated into RIC regimens the UK [2–4] while anti-thymocyte globulin is favored by others [5]. In common with many other elements, there has been no randomized comparison of these approaches. The selection of particular protocols for specific indications and patient groups remains highly idiosyncratic.

Centers providing allogeneic transplantation were recruited between January 2015 and January 2017 through direct email or telephone contact with BMT program directors, quality managers, or clinical specialist nurses. All 25 UK allogeneic centers responded with details of their protocols. Twenty-four centers reported allograft activity using sibling and unrelated donors. One center reported sibling transplantation only. Drug dose and scheduling of the major protocol groups were compiled by EBMT center number in a graphical format. Simpson’s diversity index *D* was calculated according to the formula *D* = 1 − (Σ(*n*/*N*)^2^), where *n* = number of identical protocols and *N* = total number of protocols.

**Fig. 1:**
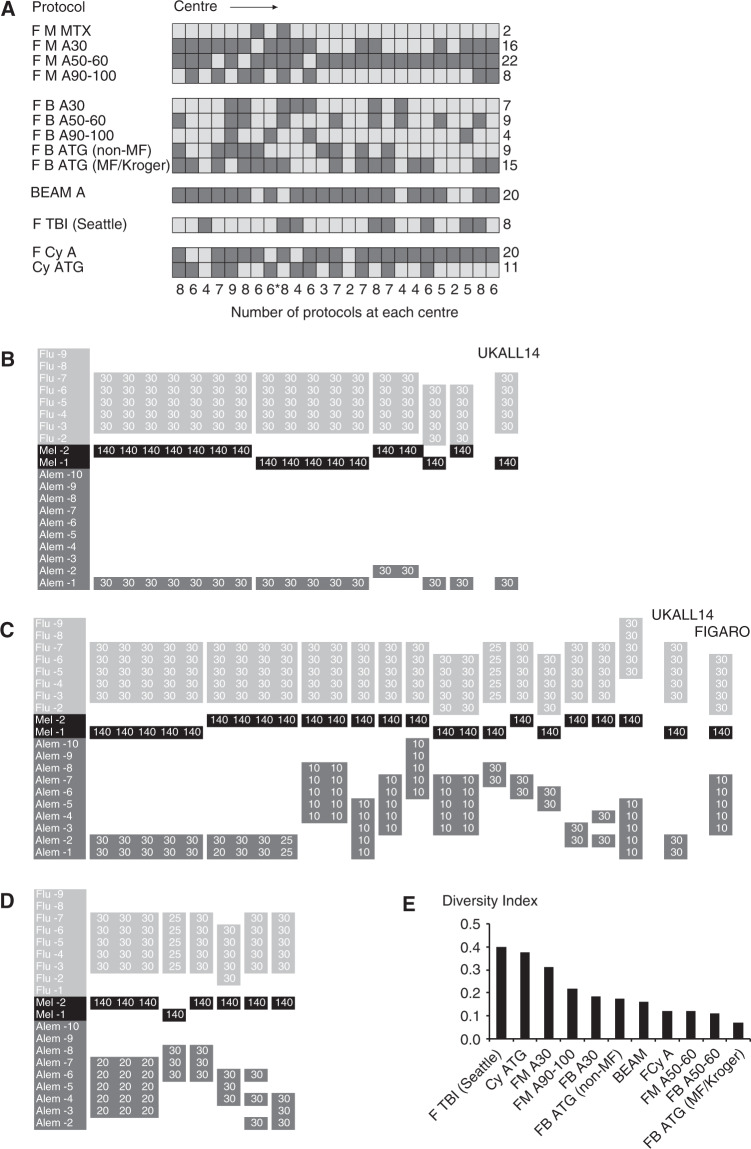
Survey of protocol diversity. **a** The range of protocols across 25 allograft centers. Dark shading denotes that the use of protocol was reported. F fludarabine, M melphalan, B busulfan, Cy cyclophosphamide, BEAM BCNU/carmustine, etoposide, ara-C, melphalan, TBI 2 Gy total body irradiation, A Alemtuzumab, ATG anti-thymocyte globulin, MTX methotrexate, MF myelofibrosis. “Kroger” based on Kroger et al. [12]; “Seattle”: based on Niederweiser et al. [6]; asterisk denotes the sibling only center. BEAM protocols include BEAM, LEAM (lomustine), LACE and F-BEAM. ATG includes Fresenius and Genzyme products. Cy ATG includes one Cy Alem protocol. Numbers following alemtuzumab denote total dose in milligrams. **b** Fludarabine melphalan and alemtuzumab 30 mg protocols. Drug, dosing, and timing of administration (day pre transplant) are defined in the vertical axis with a separate column for each center reporting a protocol. Identical protocols are grouped together across the horizontal axis. Flu fludarabine, Mel melphalan, Alem alemtuzumab. The dosing indicated is mg/m^2^ for fludarabine and melphalan and total milligram dose for alemtuzumab. Trial protocols for UKALL14 and FIGARO are indicated. **c** Fludarabine melphalan alemtuzumab 50–60 mg protocols. **d** Fludarabine melphalan alemtuzumab 90–100 mg protocols. **e** Each major protocol group was subjected to Diversity Index (DI) calculation using Simpson’s Diversity Index and arranged in decreasing order (least to most diverse).

Centers provided protocols in use for hematological malignancy including myelofibrosis and for aplastic anemia. Five major protocol groups were identified: fludarabine and melphalan (with or without alemtuzumab); fludarabine and busulfan with either alemtuzumab or ATG; Carmustine/lomustine, etoposide, cytarabine, and melphalan (BEAM) with alemtuzumab; fludarabine and TBI nonmyeloablative “Seattle” regimens [6] and protocols for aplastic anemia (Fig. 1a). Fludarabine cyclophosphamide protocols for indications other than aplastic anemia were only reported in by three centers and not included. Every center returned at least one fludarabine-based RIC protocol, the median number of protocols was 6 ranging from 2 to 9. The primary goal of the survey was to describe the diversity of protocols in use, rather than the indication for use of the protocol, the donor choice or any other ancillary factors such as the use of growth factors, and target ciclosporin level. These are likely to be important variables in outcome but it is impossible to assess their impact in the face of such protocol diversification.

Protocols were arranged into related subgroups for ease of visualization of diversity. Fludarabine melphalan protocols are shown as an example (Fig. 1b–d). A similar approach was used to describe variability in the four other main groups (Figs. S1–S4). A Diversity Index (DI) was derived using Simpson’s method to give a score between 0 (all protocols different) and 1 (all protocols identical) for each major group. Increasing DI therefore reflects greater harmonization (Fig. 1e). The most harmonized protocol was cyclophophosphamide and ATG for sibling donors in aplastic anemia (0.4). Fludarabine busulfan ATG, in which every protocol was unique, scored the lowest (0.07). A detailed description of the factors underlying variability of each protocol group is given in the Supplementary information.

The purpose of this study was to describe the extent of protocol variation, based on self-reported use of protocols by transplant centers, in order to identify potential routes to harmonization or at least a reduction of protocol diversity. Almost every parameter of a conditioning protocol was subject to variation without obvious rationale. In specific instances, erroneous reasoning was advanced, for example, transplant centers were divided almost equally about giving melphalan on day −2 or day −1 with some expressing apparently groundless concerns over residual effects on the graft when delivered at day −1. Melphalan has a very short terminal half-life of 17–75 min and proven safety in patients with end-stage renal failure, with no objective reason not to deliver the drug 24 h before stem cell infusion [7].

Much of the variation observed was due to the dose and scheduling of Alemtuzumab and ATG. The depth of T-cell depletion practised by individual transplant centers reflects notions of how much GVHD is tolerable or desirable, based on unquantifiable subjective arguments [8]. Alemtuzumab has a long in vivo half-life and the timing and fractionation of dosing is critical in its effect [9]. A commonly used dose reduction to 60 mg given as two 30 mg doses on day −2 and −1 leads to higher plasma concentrations than the 100 mg dose originally administered in five fractions from day −9 [10]. It was generally not appreciated that this “end-loaded” 60 mg schedule is not an effective dose reduction.

Busulfan-containing protocols were highly variable often due to a wide range of once daily intravenous, QDS intravenous, and even oral regimens. There was a tendency to schedule busulfan early in the regimen that is not well justified by pharmacokinetic considerations; several published studies employ this agent as late as day −2 without difficulty and with the potential advantage of fewer days of neutropenia [11]. Access to pharmacy manufacturing at the weekend remains a determinant of busulfan scheduling in the UK in more than half of transplant centers surveyed.

Fludarabine busulfan ATG protocols, intended for use in myelofibrosis, were reported by a number of centers referring to “Kroger” as a model [12]. However, all 15 were unique in some way and none reproduced the published protocol exactly. A significant source of deviation from the published protocol was the widespread use of Genzyme rabbit ATG (thymoglobulin) in place of Fresenius ATG.

Finally, a set of consensus protocols was developed. These represent the smallest number of discrete protocols that could satisfy the requirements of the majority of transplant centers. This was arrived at by two rounds of discussion and feedback from UK transplant center Directors, following presentation of the initial audit at the Anthony Nolan Annual Clinical Retreat in 2016 and 2017. The set of consensus protocols is included in the Supplementary Information for reference.

## Supplementary information

PHI report BMT SUPP V3
