# Supplementary material for: Reducing the diversity of allogeneic transplant protocols in the UK through a BSBMT Anthony Nolan Protocol Harmonization Initiative
Source: Bone Marrow Transplant. 2020 Mar 24;55(9):1840–3. doi: 10.1038/s41409-020-0870-0 (PMC7452870; doi:10.1038/s41409-020-0870-0)
Supplement: Supplementary file 1 — PHI report BMT SUPP V3 [file 41409_2020_870_MOESM1_ESM.docx]

# Details of protocol diversity

## Fludarabine melphalan protocols

Twenty-four different protocols using fludarabine and melphalan were reported **(Figure S1).** Only 2 centres had a T-replete protocol with post-grafting methotrexate and these differed in the timing of the melphalan and dosing of post-transplant methotrexate (not shown). Protocols using 50-60mg Alemtuzumab were most common (22/25 centres), followed by 30mg alemtuzumab (16 centres) and 90-100mg Alemtuzumab (8 centres). The most uniform protocol group in the study was fludarabine, melphalan and alemtuzumab 30mg which broadly corresponded with the alemtuzumab de-escalation study of Chakraverty et al. Within all fludarabine melphalan protocols there was variation in the administration of fludarabine from day -9 to -5 to day -6 to -2, melphalan either on day -2 or -1 and alemtuzumab from day -10 to -6 given as five doses to a single dose on day -1. Donor choice was indicated in a number of protocol documents but was not specifically included in the analysis. Where stated, fludarabine melphalan alemtuzumab 30mg protocols were most commonly used for sibling donors only, 50-60mg alemtuzumab protocols were used for both sibling and unrelated donors and 90-100mg alemtuzumab protocols were used for sibling, unrelated and mismatched unrelated donors. Centres reporting an indication stated that fludarabine melphalan was used for transplantation of patients with indications including lymphoma, acute lymphoblastic leukaemia, myelodysplasia and acute myeloid leukaemia. 14/25 centres reported a BEAM protocol in addition to fludarabine melphalan, providing a choice of two protocols for lymphoma. 18/25 centres reported fludarabine busulfan as an alternative for myelodysplasia and acute myeloid leukaemia. 7/25 centres did not use fludarabine busulfan (except for myelofibrosis) and presumably therefore employed fludarabine melphalan for myelodysplasia and acute myeloid leukaemia. Two centres had arrangements for starting fludarabine as an outpatient, one using oral dosing of 50mg/m^2^. Transplant regimens were specified in two trials at the time of audit; UKALL14 (EudraCT: 2009-012717-22) and FIGARO (EudraCT: 2012-005538-12). UKALL14 specified two of the most commonly used protocols for fludarabine melphalan alemtuzumab 30mg/60mg but FIGARO included a variant of fludarabine melphalan alemtuzumab 50mg practised by only 2 centres (and identified as their FIGARO protocol). A ciclosporin dose of 3mg/kg was specified by 11 centres. In addition to alemtuzumab, one centre used mycophenolate mofetil (MMF) and one centre used post-transplant methotrexate (MTX). Both of these employed alemtuzumab early between day -10 and -6. A third centre with early alemtuzumab scheduling (day -8 and -7) used ciclosporin monotherapy. One centre specified a reduced 110mg dose of melphalan for patients with reduced creatinine clearance. Most centres did not include this information on their protocols but reported that fludarabine and melphalan were both reduced in proportion to creatinine clearance in accordance with local pharmacy policy.

### Figure S1. Fludarabine melphalan protocols

Protocol drug, dosing and timing of administration (day pre-transplant) defined in the vertical axis with a separate column for each centre reporting a protocol. Identical protocols are grouped together across the horizontal axis. Abbreviations: Flu: fludarabine; Mel: melphalan; Alem: alemtuzumab. The dosing indicated is milligram/m^2^ for fludarabine and melphalan and total milligram dose for Alemtuzumab. Trial protocols for UKALL14 and FIGARO are indicated.

**A.** Fludarabine melphalan alemtuzumab 30mg protocols

**B.** Fludarabine melphalan alemtuzumab 50-60mg protocols

**C.** Fludarabine melphalan alemtuzumab 90-100mg protocols


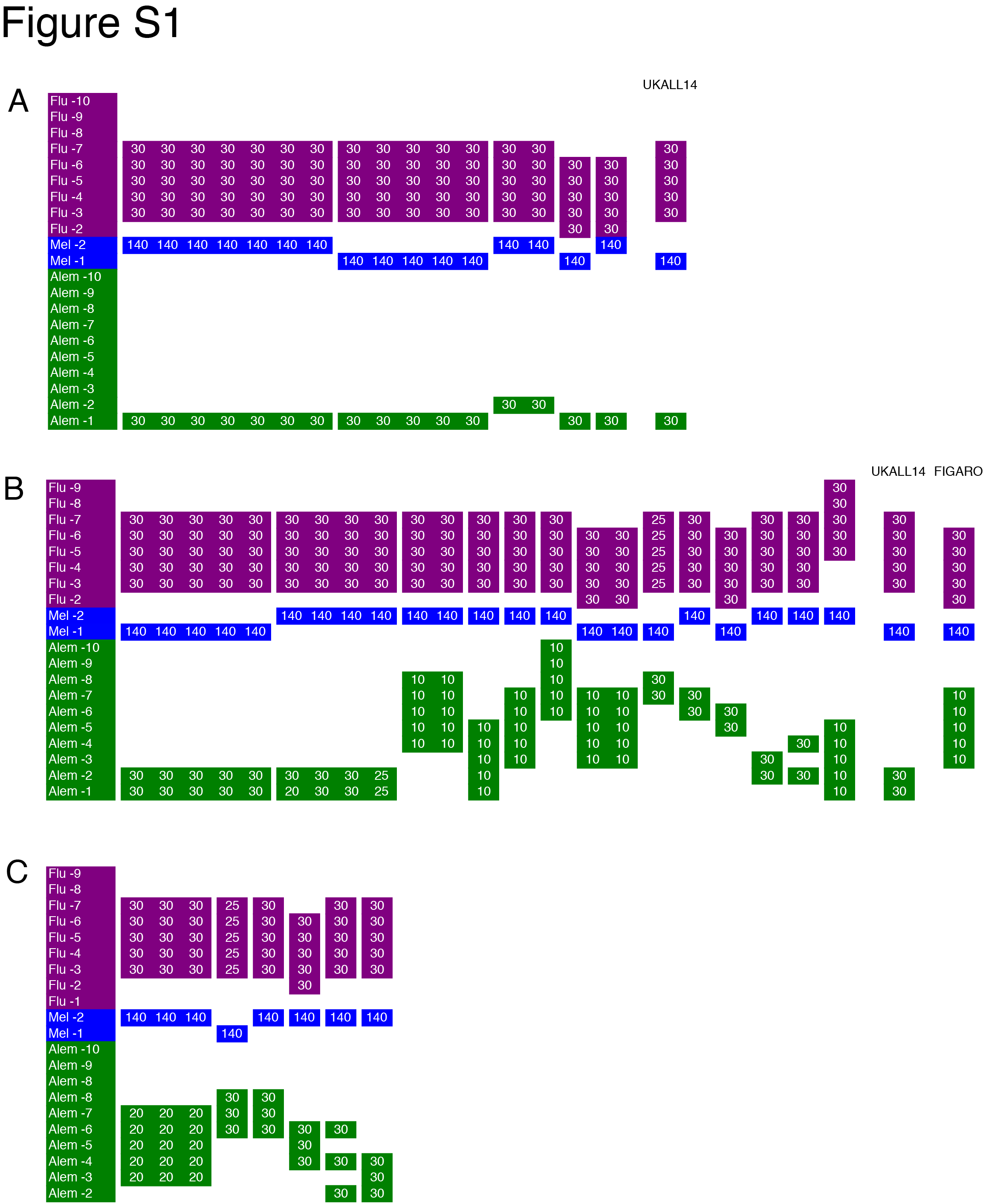


## Fludarabine busulfan protocols

Fludarabine busulfan protocols were divided into three groups: non-myelofibrosis protocols with Alemtuzumab (**Figure S2A**) or with ATG (**Figure S2B**) and myelofibrosis protocols (**Figure S2C**). Non-myelofibrosis protocols were reported by 13/25 centres using alemtuzumab and 9/25 using ATG. Overall, fludarabine busulfan protocols were the most diverse. Of 28 reported protocols using alemtuzumab or ATG, 26 were unique and only 2 protocols were each common to 2 centres. Within 3 discrete protocols, additional variations of busulfan or ATG dose were annotated. The most prevalent regimens, using 50-60mg alemtuzumab or ATG, were reported by 10 and 8 centres respectively. One centre reported fludarabine busulfan and MTX (not shown). Variations between protocols included: commencement of fludarabine from day -9, -to day -5; total dose, scheduling and route of administration of busulfan; total dose and scheduling of alemtuzumab or ATG; and use of post-grafting methotrexate or MMF. Variation in the scheduling of busulfan was partly due to the availability of pharmacy at weekends, such that protocols intended for use with the FIGARO trial ranged from administration of two doses on early on day -6 and -5 to late on day -3 and -2. Most protocols adopted single daily IV busulfan at 3.2mg/kg without therapeutic monitoring of drug levels. The most common dose of ATG was 2.5mg/kg of Genzyme rabbit ATG (Thymoglobulin) on days -2 and -1 although the total dose used ranged from 3mg to 12mg per kg. All non-myelofibrosis ATG-containing regimens used either post-grafting methotrexate or MMF, the latter specified as 1g three times daily in 8/9 protocols. Ciclosporin dose was indicated in 9 protocols as 3mg/kg. Fludarabine busulfan protocols were mostly but not exclusively used for myelodysplasia and acute myeloid leukaemia. Fludarabine busulfan ATG protocols intended for use in myelofibrosis were reported by 15/25 centres. A number referred to ‘Kroger’ as a model but all 15 were unique in some way and none reproduced the published protocol exactly. A significant source of deviation from the published protocol was the use of Genzyme rabbit ATG (thymoglobulin) in place of Fresenius ATG, retained by only 3/15 centres. None of these, however used the higher 20mg dose for unrelated donors. 7/15 protocols started at day -9 rather than day -7 and many used later scheduling of busulfan between day -5 and day -3, rather than -7 to -5 as described by Kroger. Most but not all employed either methotrexate or MMF as post-grafting GVHD prophylaxis. Where specified, the dose of corticosteroids administered with ATG varied substantially from 0.5mg/kg to 2mg/kg equivalent to a total dose range 1.5mg/kg to 8mg/kg (not shown). 10/25 centres did not report a protocol specifically for myelofibrosis suggesting that alternative conditioning was being used for this indication.

### Figure S2. Fludarabine busulfan protocols

Protocol drug, dosing and timing of administration (day pre-transplant) defined in the vertical axis with a separate column for each centre reporting a protocol. Identical protocols are grouped together across the horizontal axis. Abbreviations: Flu: fludarabine; Bu: busulfan; Alem: Alemtuzumab; ATG: anti-thymocyte globulin (black text for Fresenius, white text for Genzyme); MTX: methotrexate; MMF: mycophenolate mofetil. The dosing indicated is fludarabine: milligram/m^2^; bulsulfan: milligram/kg; Alemtuzumab: total milligram dose; methotrexate: milligram/m^2^. Trial protocols for FIGARO are indicated.

**A.** Fludarabine busulfan alemtuzumab protocols. Busulfan: paretheses (3.2) indicates optional reduction for older patients or comorbidity; asterisk * divided between four intravenous doses; ** divided between 4 oral doses. Methotrexate: *** +1 day compared with vertical axis label.

**B.** Fludarabine busulfan ATG protocols for non-myelofibrosis. Busulfan: ** divided between 4 oral doses. ATG parentheses (2.5) additional dose reserved for unrelated donors.

**C.** Fludarabine busulfan ATG protocols for myelofibrosis. Busulfan: parentheses: dose omitted in over 60 years; * divided between four intravenous doses; ** divided between 4 oral doses; # 2 x 1.6mg doses. Mycophenolate mofetil ## used only with unrelated donors.


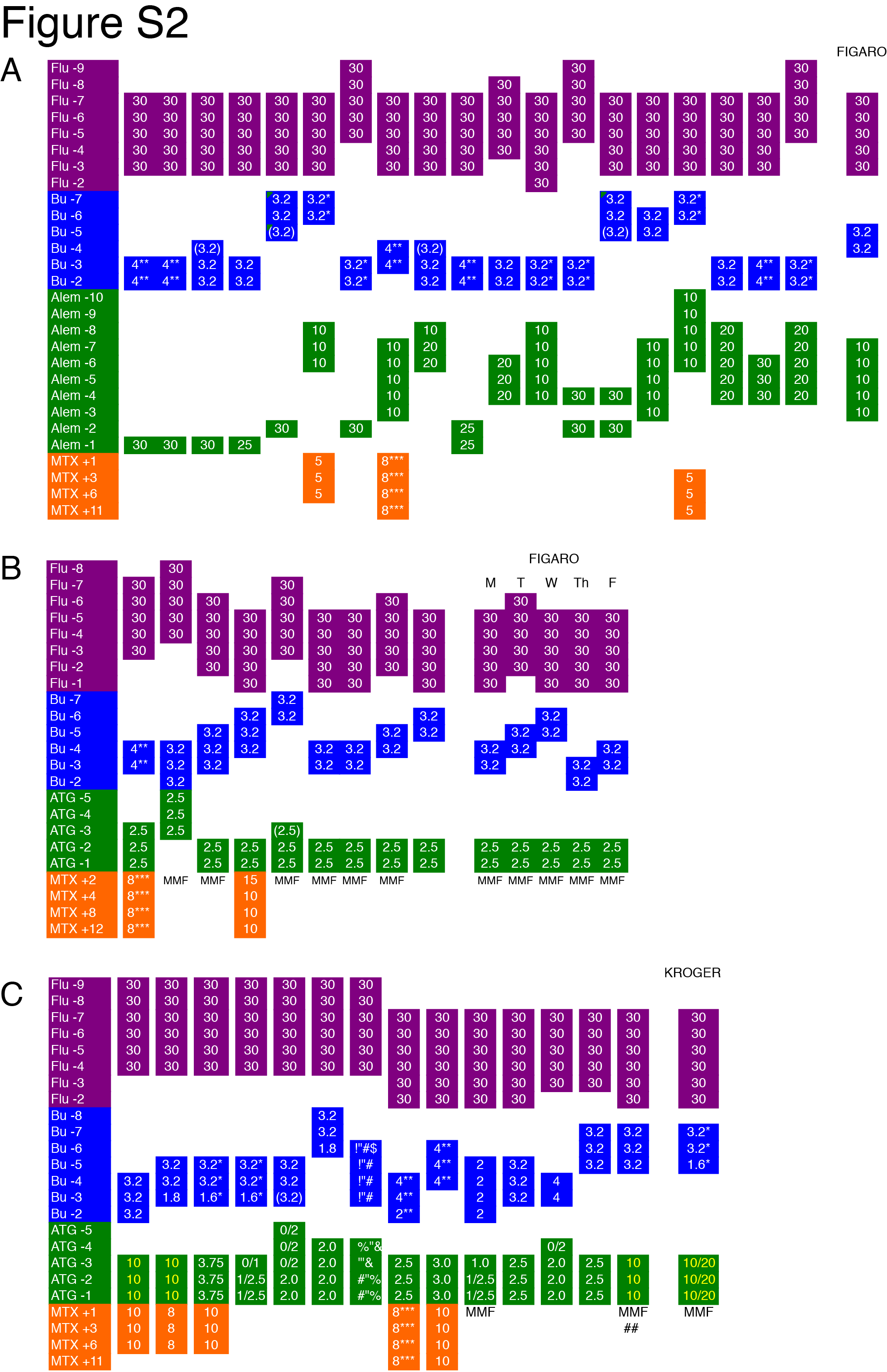


### BEAM and Seattle protocols

Many centres (19/25) reported a BCNU/Carmustine, Etoposide, Ara-C and Melphalan ‘BEAM’ protocol for use with lymphoma (**Figure S3A**). Recent difficulties of access to BCNU led to alternative Lomustine or LEAM protocols, considered as a single protocol group with BEAM. These protocols were generally low in diversity and were based on a single backbone starting either day -7 or day -6. 15/25 centres started on day -6 with melphalan given on day -1. Almost half of these (7/15) used the same 5 x 10mg alemtuzumab schedule. Ciclosporin was given at 3mg/kg where specified. Two centres specified fludarabine 30mg/m^2^ on days -9 to -7 (3 doses) for unrelated donors (not shown) and 3 centres had alternate 50/100 alemtuzumab dosing for siblings and unrelated donors, respectively. Fludarabine 2Gy total body irradiation (Seattle) protocols were reported by 8/25 centres for use in patients with multiple myeloma, chronic lymphocytic leukaemia and frail patients with other indications (**Figure S3B**). All centres used MMF and ciclosporin at higher dose (5mg/kg); in addition, two centres used 4 doses of methotrexate. Fludarabine was consistent except one centre used oral dosing; TBI was either given on day -1 or the day of transplantation.

### Figure S3. BEAM and Seattle protocols

Protocol drug, dosing and timing of administration (day pre-transplant) defined in the vertical axis with a separate column for each centre reporting a protocol. Identical protocols are grouped together across the horizontal axis. Abbreviations: Car: carmustine (white text); Lo: lomustine (black text); Etop: etoposide; Ara-C: cytosine arabinoside; Mel: melphalan; Alem: Alemtuzumab; Flu: fludarabine; TBI: 2Gy total body irradiation; MTX: methotrexate. The dosing indicated is milligram/m^2^ for BCNU/carmustine, lomustine, etoposide, ara-C, melphalan, fludarabine and methotrexate; total milligram dose for Alemtuzumab; Gy for TBI.

**A.** BEAM or LEAM protocols. Alemtuzumab: paretheses (30) indicates dose used only for unrelated donors asterisk *increased to 20mg for unrelated donors **increased to 2 x 25 mg for unrelated donors.

**B.** Fludarabine 2Gy total body irradiation protocols (Seattle). Fludarabine: * oral dose.


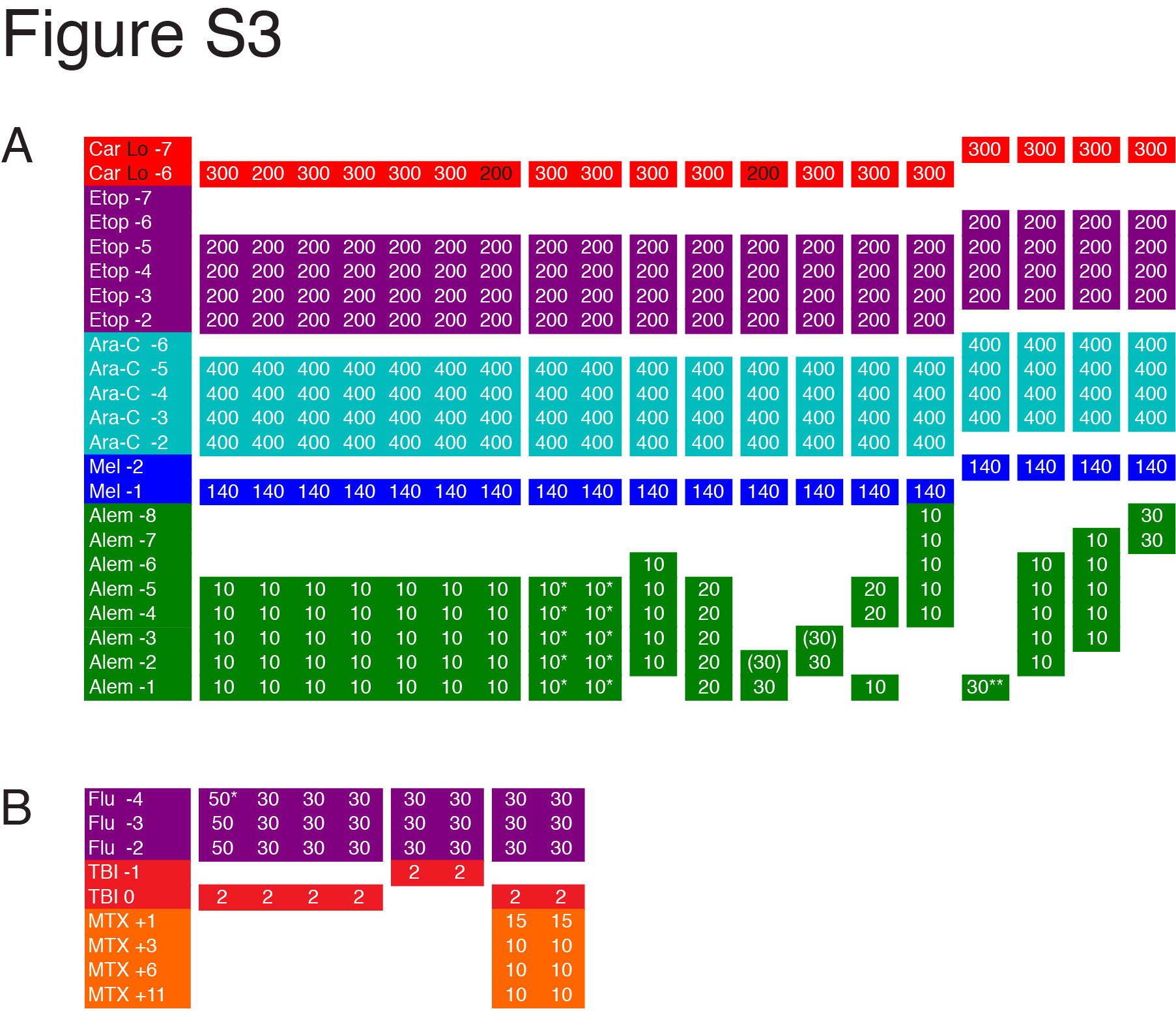


### Aplastic anaemia protocols

Nearly all centres (23/25) reported at least one aplastic anaemia protocol (**Figure S4**). Two main groups emerged: alemtuzumab-containing protocols used in 20 centres with both sibling and unrelated donors (fludarabine, cyclophosphamide and alemtuzumab); and Genzyme ATG protocols, used in 10 centres for sibling donors (cyclophosphamide and ATG). There was considerable variation in the timing of all drugs with the former but in the latter, 6/10 protocols were identical. Three centres specified ATG alternatives for their alemtuzumab protocols, for use with CD52-negative PNH clones. Three centres offered alemtuzumab in place of ATG for their cyclophosphamide-based sibling protocols for patients intolerant of ATG. Four centres retained the option to add 2Gy TBI at day -1 to their fludarabine, cyclophosphamide and alemtuzumab protocol for unrelated donors and one centre with their cyclophosphamide and ATG protocol. All ATG-containing protocols specified post-transplant methotrexate. Methyl prednisolone was included to cover ATG and Alemtuzumab administration by some centres at either 1 or 2 mg/kg per dose. Ciclosporin GVHD prophylaxis, where specified, was 5mg/kg per day.

### Figure S4. Protocols for Aplastic anaemia

Protocol drug, dosing and timing of administration (day pre-transplant) defined in the vertical axis with a separate column for each centre reporting a protocol. Identical protocols are grouped together across the horizontal axis. Abbreviations: Flu: fludarabine; Cy: cyclophosphamide; TBI: total body irradiation; Alem: almetuzumab; ATG: antithymocyte globulin (Genzyme); MTX: methotrexate. The dosing indicated for fludarabine, cyclophosphamide ‘300’ and methotrexate is milligram/m^2^; alemtuzumab ’10, 15, 25, 30’ is total milligram dose; alemtuzumab ‘0.2’, ATG and cyclophosphamide 50 and ATG is milligram/kg for; TBI is Gy.

**A.** Fludarabine cyclophosphamide alemtuzumab protocols. TBI: parentheses indicate optional TBI. Alemtuzmab: parentheses indicate additional doses for unrelated donors only; *ATG-containing alternative recommended for patients with CD52-negative clone.

**B.** Cyclophosphamide ATG protocols. TBI: parentheses indicate optional TBI. MTX: parentheses indicate additional dose for PBSC grafts; **+1 day compared with vertical axis label.


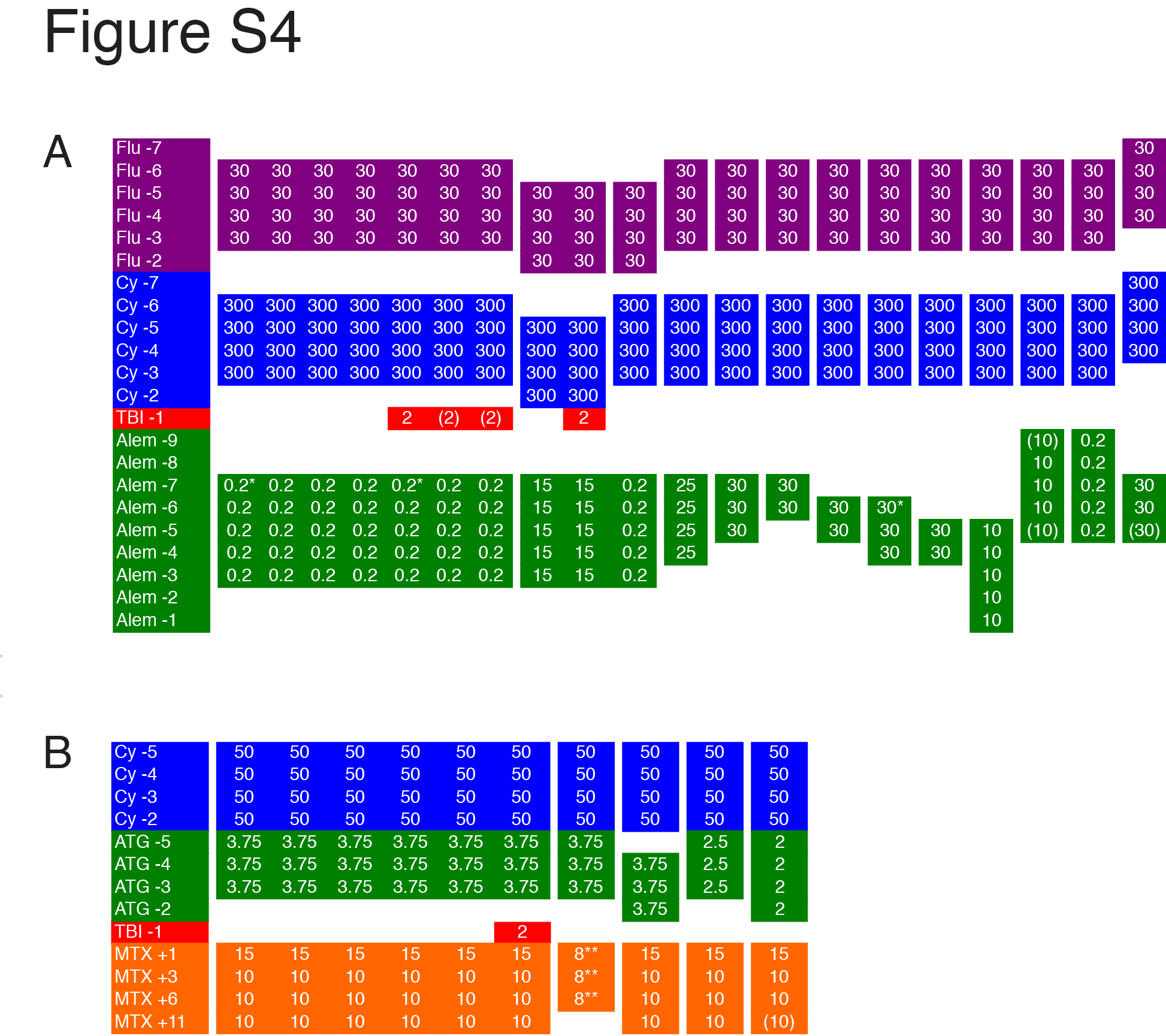


## Consensus protocols

### 1. Fludarabine melphalan alemtuzumab

Fludarabine mg/m^2^

Melphalan mg/m^2^

Alemtuzumab total mg

### 2. Fludarabine busulfan alemtuzumab

Fludarabine mg/m^2^

Busulfan mg/kg

Alemtuzumab total mg

### 3. Fludarabine busulfan Genzyme ATG

Fludarabine mg/m^2^

Busulfan mg/kg

ATG mg/kg

### 4. Fludarabine busulfan Genzyme ATG ‘Kroger’ for myelofibrosis

Fludarabine mg/m^2^

Busulfan mg/kg

ATG mg/kg


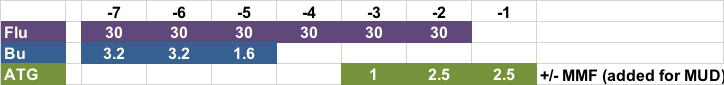


### 5. BEAM alemtuzumab

Carmustine/Lomustine mg/m^2^

Etoposide mg/m^2^

Ara-C mg/m^2^

Melphalan mg/m^2^

Alemtuzumab total mg

### 6. Seattle non-myeloablative

Fludarabine mg/m^2^

TBI Gy

MTX mg/m^2^

### 7. Aplastic

Fludarabine mg/m^2^

Cyclophosphamide mg/m^2^

Alemtuzumab mg/kg

TBI Gy
